# Neurotechnology Governance in the United States: Gaps and Opportunities

**DOI:** 10.1111/bioe.70062

**Published:** 2025-11-28

**Authors:** Laura Y. Cabrera, Nia Evereteze, Emily G. Shank, Jennifer K. Wagner, Michele Mekel, Jennifer B. McCormick, Megan S. Wright

**Affiliations:** ^1^ Department of Engineering Science and Mechanics, Rock Ethics Institute, Huck Institutes of the Life Sciences, Philosophy, and Center for Neural Engineering The Pennsylvania State University; University Park Pennsylvania USA; ^2^ Penn State Law; University Park Pennsylvania USA; ^3^ Neuroethics Lab The Pennsylvania State University; University Park Pennsylvania USA; ^4^ School of Engineering Design and Innovation, Department of Anthropology, Department of Biomedical Engineering, Institute for Computational and Data Sciences, Huck Institutes of the Life Sciences, Rock Ethics Institute, and Penn State Law The Pennsylvania State University; University Park Pennsylvania USA; ^5^ Bioethics Program, College of the Liberal Arts; Rock Ethics Institute; Humanities Department, College of Medicine; Penn State Law The Pennsylvania State University, University Park Pennsylvania USA; ^6^ Department of Humanities and Biomedical Sciences Program Faculty, College of Medicine The Pennsylvania State University Hershey Pennsylvania USA; ^7^ Penn State Dickinson Law, Department of Sociology, Rock Ethics Institute, and Bioethics Program The Pennsylvania State University, University Park Pennsylvania USA; ^8^ Department of Public Health Sciences and Department of Humanities, College of Medicine The Pennsylvania State University Hershey Pennsylvania USA; ^9^ Adjunct Assistant Professor of Medical Ethics in Medicine Weill Cornell Medicine New York New York USA; ^10^ Visiting Lecturer in Law, Yale Law School New Haven Connecticut USA

## Abstract

Neuroscience's accelerating advances have reached a pivotal point in the study of the human brain, including neurotechnologies capable of recording large amounts of data and acting with greater precision. However, the use of neurotechnology has raised a number of ethical, legal, and social implications (ELSI). To that end, sufficiently robust policy and governance structures must be considered. To date, no published review of United States policies governing neuroscience and neurotechnology exists. To address this, we review US polices and various ethical frameworks overseeing neuroscience and neurotechnology. This policy review highlights where gaps in neuroscience and neurotechnology policy and governance might exist. Overall, our review shows that “soft policies” make up the present‐day US neurotech‐governance universe at the federal level, with neurodata specific state‐legislation emerging. The included analysis can aid researchers, technology developers, neuroethicists, research ethicists, legal scholars, and others in facilitating ethically and socially responsible implementation of neuroscience and neurotechnology as they move from “bench to bedside and beyond.”

## 1 Introduction

Neuroscience's accelerating advances have reached a pivotal point in the study of the human brain, including neurotechnologies capable of recording large amounts of data and acting with greater precision. Coupled with the fact that neuroscience and neurotechnology are applied in a wide variety of domains, predominantly in medicine, but extending to other applications including law, employment, and entertainment [1], neuroscience and neurotechnology have the potential to impact our daily lives and society in unprecedented ways: some positive and some negative. In the United States, advances in neuroscience and neurotechnology have been catalyzed as the result of the Brain Research through Advancing Innovative Neurotechnologies (BRAIN) Initiative, launched in 2013 by then President Barack Obama as one of the Administration's Grand Challenges. By unraveling the brain's many mysteries, the BRAIN Initiative intended to do for neuroscience what the Human Genome Project did for genomics [2].

However, along side the promises of advanced neurotechnology, its use has raised a number of ethical, legal, and social implications (ELSI), including: safety and effectiveness [3, 4]; privacy [5, 6]; justice [7, 8]; invasiveness [9, 10]; and alteration of self‐related characteristics, such as personality, identity, autonomy, agency, and authenticity [11, 12].

Sufficiently robust policy and governance structures must be considered in facilitating ethical and socially responsible research, knowledge translation, and technological deployment within neuroscience and neurotechnology. Relevant policies may include legally binding legislation at the federal and/or state level(s), as well as international instruments (such as treaties), to define permitted and/or prohibited conduct. However, policy instruments can also include non‐legislative mechanisms or “soft policy,” such as guidelines and recommendations [13]. Standards enacted via sanctioned legislative or legal mechanisms (e.g., regulations, court decisions) carry the weight and authority of law, whereas soft policies are nonbinding and utilize moral persuasion to influence conduct.

To date, no published review of US policies—legislative or otherwise—governing neuroscience and neurotechnology exists.^1^ We believe such a review is crucial in identifying legislative and regulatory gaps. To address this, we review US policies and various ethical frameworks overseeing neuroscience and neurotechnology, and then we map and analyze the current principles and recommendations on ethical neurotechnology and related governance frameworks.

Our review demonstrates that, for the most part, “soft policies” make up the present‐day US federal‐level neurotech‐governance universe, with neurodata specific state‐legislation emerging. Despite calls for the implementation of robust policy and governance structures by national‐ and international‐level professional organizations (e.g., IEEE BRAIN, IEEE SA), academic groups [14], private companies, research institutions, and non‐profit entities [15, 16], current neurotech governance in the United States consists predominantly of guidelines, recommendations, position papers, frameworks, and standards for ethical neurotechnology that these bodies themselves have established. By contrast, in the international arena, Chile amended its constitution in 2021 to protect “mental integrity,” and other countries (such as Mexico, Brazil, Costa Rica, Colombia, Uruguay, and Argentina) have begun considering neurotech legislation (see, e.g. [17]). In the United States, however, legislative efforts have been occurring primarily at the state level, with several states considering bills to explicitly protect so‐called “neural data.”^2^ For example, in April 2024, Colorado amended the state's Privacy Act explicitly to protect “neural data” [18], making it the first in the country to do so. As of April 2025, Colorado was joined by California in enacting state legislation to overtly protect “neural data” rights.

Our national level policy analysis and mapping results reveal that elected officials creating public neuroscience and neurotechnology policy, as well as creators of private policies (such as advisors and think tanks), share concerns about integration, capacity building and strengthening, guidance and oversight, privacy and security, safety and wellbeing, justice, responsibility, autonomy, and transparency. This focused policy review highlights where gaps in neuroscience and neurotechnology policy and governance might exist. The included analysis, while limited in scope, can aid researchers, neuroethicists, research ethicists, and legal scholars in facilitating ethically and socially responsible implementation of neuroscience and neurotechnology as they move from “bench to bedside and beyond.”

## 2 Methods

Our review focused on policies within the United States that appeared since the launch of the BRAIN Initiative in 2013 and that were motivated as a result of the advances in neurotechnology. To achieve that goal we relied on publicly accessible government and scientific foundation websites—active and archived—to locate relevant materials. Specifically, the websites examined included those listed in Table 1.

**Table 1:** Websites of organizations linked to the BRAIN Initiative that were searched.

Organization acronym	Full name of organization
FDA	Food and Drug Administration
NIH	National Institute of Health
NSF	National Science Foundation
DARPA	Defense Advanced Research Projects Agency
DoD	Department of Defense
IARPA	Intelligence Advanced Research Projects Activity
NCCIH	National Center for Complementary and Integrative Health
NEI	National Eye Institute
NIA	National Institute of Aging
NIAAA	National Institute on Alcohol Abuse and Alcoholism
NIBIB	National Institute of Biomedical Imaging and Bioengineering
NICHD	National Institute of Child Health and Human Development
NIDA	National Institute on Drug Abuse
NIDCD	National Institute on Deafness an dOther Communication Disorders
NIMH	National Institute of Mental Health
NINDS	National Institute of Neurological Disorders and Stroke
AI	Allen Institute
HHMI	Janelia/Howard Hughes Medical Institute
KF	Kavli Foundation
SIB	Salk Institute for Biological
SF	Simons Foundation
INS	International Neuroethics Society
ABC	American Brain Coalition
IEEE	Institute of Electrical Electronic Engineers
DF	Dana Foundation

In addition, to ensure a wide net, we also used WestLaw, a database of primary and secondary legal sources, including statutes, regulations, and caselaw. In our searches, we included documents that were: (a) policies, enacted legislation (but not merely proposed legislation), regulations, and legal pronouncements (established between 2013 and April 2024) related to neurotechnology; (b) legally enforceable policies of BRAIN‐Initiative funding organizations, including governmental agencies (see Table 1 for more details), as well as non‐profits and think tanks (i.e., the Allen Institute for Brain Science, the Kavli Foundation, and the Howard Hughes Medical Institute); (c) BRAIN Initiative policies that are not limited to neurotechnology, but, rather, are inclusive of neurotechnologies; and (d) consensus statements by professional organizations directly related to neurotechnology. We excluded documents that were: (a) BRAIN‐Initiative funding mechanism descriptions; (b) non‐neurotechnology‐related legislation, regulation, policy, and caselaw; (c) nonbinding gray papers; (d) book chapters; (e) press releases; and (f) documents focused on international governance.

We used as search terms *neurotechnology* with *policy* and *guidance*. While this may be narrow, we opted for simple, specific search terms to ensure precision and relevance. Every link identified in our search results was followed and screened for neurotechnology policies, guidelines, or recommendations, or other soft‐policy mechanisms. After removing duplicates, the team screened the initial set of potential documents, resulting in only eight documents (see Table 2).

**Table 2:** Description of documents identified and analyzed.

Title	Year	Source	Authority	Type	Area of focus
*Neurotechnology Future Study*	2013	A Potomac Institute for Policy Studies Report	An independent, not‐for‐profit policy research institute that develops meaningful science and tech policy options; product of a convened study group	Roadmap outlining key research areas and technologies needed to move neurotechnology forward, including a set of recommendations	Roadmap for the progression of neurotechnologies; identify potential federal investments that could change or advance the progression of neurotechnologies; and identify and analyze the potential social, ethical, and political implications of neurotechnologies
*Gray Matters: Integrate Ethics and Science through Education at All Levels, Volume 1*	2014	Presidential Commission for the Study of Bioethical Issues	Advisory panel (“nation's leaders in medicine, science, ethics, religion, law, and engineering”); not legally binding/enforceable	Recommendations about the integration of ethics into neuroscience research and its applications	Importance of integrating ethics and neuroscience early and throughout the research endeavor; four examples provided: neuroimaging and brain privacy, dementia, personality, and changed preference; cognitive enhancement and justice; and deep brain stimulation research and the ethically difficult history of psychosurgery
*Gray Matters: Topics at the Intersection of Neuroscience, Ethics, and Society, Volume 2*	2015	Presidential Commission for the Study of Bioethical Issues	Advisory panel (“nation's leaders in medicine, science, ethics, religion, law, and engineering”); not legally binding/enforceable	Recommendations about ethical neuroscience research and application thereof	Cognitive enhancement, consent capacity, neuroscience and legal proceedings
*Neuroethics Guiding Principles for the NIH BRAIN Initiative*	2018	NIH working group known as “Neuroethics Working Group of the National Institutes of Health (NIH) Brain Research through Advancing Innovative Neurotechnologies (BRAIN) Initiative	Advisory working group (“nation's leaders in medicine, science, ethics, religion, law, and engineering”); not legally binding/enforceable	Recommendations focused on BRAIN Initiative	Assessment of risks; confidentiality; equity considerations
*The BRAIN Initiative and Neuroethics: Enabling and Enhancing Neuroscience Advances for Society*	2019	Neuroethics subcommittee (BRAIN neuroethics subgroup or BNS) of Advisory Committee to NIH Director	Subcommittee of an advisory committee to NIH Director about BRAIN Initiative; not legally binding/enforceable	Recommendations about including neuroethics research in neuroscience research funded by NIH BRAIN Initiative (integration rather than parallel)	NIH BRAIN Initiative Priority Areas
*Brain‐Computer Interfaces: U.S. Military Applications and Implications*	2020	RAND Corporation	Non‐profit global policy think tank, research institute, and public‐sector consulting firm	Recommendations	Brain‐computer interfaces in the military
*BRAIN 2.0 Neuroethics: Enabling and Enhancing Neuroscience Advances for Society*	2023	Neuroethics subcommittee (BRAIN neuroethics subgroup or BNS) of Advisory Committee to NIH Director	Subcommittee of an advisory committee to NIH Director about BRAIN Initiative; not legally binding/enforceable	Recommendations about including neuroethics research in neuroscience research funded by NIH BRAIN Initiative	NIH BRAIN Initiative Priority Areas
*Amendment to Colorado's Privacy Act*	2024	General Assembly of the State of Colorado	State Legislation, legally enforceable	State Legislation	Expands the Colorado Privacy Act to include the protection of the privacy of “neural data”

We then employed thematic content analysis to compare different areas of policy or recommendation foci within these eight sources [19, 20]. Each document was independently coded by two researchers (ES and NE), supplemented by coding of assigned sources by the other co‐authors. Another member of the team (LC) triangulated the coding schemes of the two independent coders with some a priori codes used in similar literature to create a revised codebook. The resulting codebook was then revised by the whole team to finalize themes and their descriptions. The following were the themes that emerged from the analysis: integration, capacity building, privacy and security, guidance and oversight, safety and wellbeing, autonomy, responsibility, justice, and transparency. We also coded each document based on source, authority, type, and content area(s).

## 3 Results

The majority of the eight documents identified contained ethical guidelines, recommendations, and other soft‐policy provisions. The remaining item was a state‐level legislative amendment governing neurotechnology‐related data (Table 2). Compared to the international landscape, which has witnessed an uptick of such materials over the course of the past decade, we found only a slow emergence of such materials in the United States during the same timeframe.

Data by type of authority indicate that most identified policy documents contain only soft‐governance mechanisms and, similarly, that most identified documents provide ethical recommendations addressed to multiple stakeholder groups, including researchers, institutional review boards (IRBs), NIH leaders, and advisory bodies. Moreover, all but one of the identified documents [21] broadly address neuroscience advances and neurotechnology. Data broken down by type of issuing organization show that most documents were produced by governmental advisory bodies (*n* = 5/8; 62.5%), followed by non‐profit organizations (*n* = 2/8; 25%). For example, the *Gray Matters* reports, drafted under the aegis of the then‐existing Presidential Commission for the Study of Bioethical Issues, are a clear example of reports generated by government advisory bodies. Others were linked to advisory bodies/committees specific for the BRAIN Initiative. Two documents were from a non‐profit policy research institute and a non‐profit global policy think tank, respectively (see Table 2).

### 3.1 Summaries of Policy Documents Reviewed

*Neurotechnology Futures Study* (*NFS*): This report by the Potomac Institute for Policy Studies discusses the future development of neurotechnology and examines the ELSI concerns that potentially could arise as neurotechnology advances. The report includes a roadmap with dual tracks that contemplate the two key research areas and technologies allowing for the advance of neurotechnology. Track 1 focuses on fundamental science and the related ELSI. Track 2 focuses on technologies, applications, and the related ELSI that might result. The report identifies key areas in which federal and state government investment is critical and could impact neurotechnology development and connected ELSI [22].

*Gray Matters 1: Integrate Ethics and Science through Education at All Levels, Volume 1* (*GM1*): This report by the former Presidential Commission for the Study of Bioethical Issues begins with a brief overview of neuroscience. It transitions to an introduction of ethics integration and the role ethics plays in neuroscience development. The report advocates for ethics integration early and explicitly in all research endeavors. It also encourages evaluation of existing and innovative approaches to ethics integration. Additionally, the report calls for consolidating ethics and science at all levels of education. Finally, the report recommends that ethical perspectives be unambiguously represented on advisory and review boards [23].

*Gray Matters 2: Topics at the Intersection of Neuroscience, Ethics, and Society, Volume 2* (*GM2*): This document, a follow up to the previous report by the former Presidential Commission for the Study of Bioethical Issues, offers 14 recommendations about cognitive enhancement, research consent capacity, and neuroscience and law. Of particular importance are the recommendations related to the necessity for legal clarity regarding ethical research inclusion of individuals with impaired decision making. While some nonbinding, older federal agency guidance exists, this report calls for direct legal guidance in this arena [24].

*Neuroethics Guiding Principles for the NIH BRAIN Initiative* (*NIH P*): The document by the Neuroethics Working Group of the NIH articulates the necessity of focusing on ethical considerations as neurotechnology develops, asserting two main principles: (1) advancing neurotechnology is an ethical imperative due to the significant economic burden and profound distress caused by brain‐related diseases and disorders; and (2) neuroethics is essential to the progress of neuroscience and neurotechnology research, as ELSI integration can serve as a guidepost for ensuring that neuroscience advancement supports human well‐being. Through recommendations, the authors advocate for responsible continued development of neurotechnology [25].

*The BRAIN Initiative and Neuroethics: Enabling and Enhancing Neuroscience Advances for Society* (*NIH EENAS*): The document by the Neuroethics subcommittee of the Advisory Committee to the NIH Director provides an overview of the BRAIN Initiative, highlighting the rapid progress of BRAIN Initiative‐funded research. Recognizing the unique nature of BRAIN Initiative‐funded research and its implications for society, the document underscores the importance of integrating neuroethics principles and approaches to effectively navigate ethical challenges. It recommends enhanced collaboration among neuroscientists and neuroethicists, dedicating resources to neuroethics research, and establishing guidelines for data management. Additionally, it highlights the need to address neuroethics implications in diverse contexts beyond biomedicine [26].

*Brain‐Computer Interfaces: US Military Applications and Implications* (*BCI Report*): This report by the RAND Corporation, considers the potential roles and risks of brain‐computer interface (BCI) technologies related to military applications. The report relies on literature reviews and a meeting of subject matter experts. The report recommends that DoD expand its analysis to illuminate operational relevance and risks; address the trust deficit and how it will impact military relationships; collaborate and anticipate work with the private sector; and plan for institutional implications [21].

*BRAIN 2.0 Neuroethics: Enabling and Enhancing Neuroscience Advances for Society* (*BRAIN 2.0*): The report by the Neuroethics Subcommittee of Advisory Committee to the NIH Director reviews priority areas identified in the BRAIN 2025 strategic plan and incorporates updates from the broader BRAIN Working Group 2.0. It characterizes the neuroethical implications that might arise as BRAIN Initiative investments produce new tools and/or as those tools are applied to advancing the goals of the NIH BRAIN Initiative. Among the topics discussed are considerations for performing neuroscience research involving human participants, developing neuroethics research opportunities, and integrating neuroethics and neuroscience [27].

*Amendment to the Colorado Privacy Act/H.B. 24‐1058* (*CO 24*): This statutory amendment by the General Assembly of the State of Colorado expands the definition of “sensitive data” in Colorado's Privacy Act to include, among other things, “neural data,” as data obtained from measuring the activity of a person's central or peripheral nervous systems, which can be processed by or with the aid of a device. Taking a “neurorights” approach, this amendment treats neural data as particularly sensitive, individualistic, and potentially subject to unique abuses [18].

### 3.2 Overarching Themes

Nine themes emerged from analyzing the eight documents. These themes are listed by the number of sources in which they were featured (Table 3). Below, we have described each theme, starting with integration—the theme that emerged most frequently—and ending with transparency—the theme that appeared least frequently. Integration, capacity building, and guidance and oversight were referenced in six of eight sources.

**Table 3:** Principles and themes identified across documents.

	NFS	GM 1	GM 2	NIH P	NIH EENAS	BCI Report	BRAIN 2.0	CO 24
Integration	x	x	x		x	x	x	
Capacity building	x	x	x	x	x		x	
Guidance and oversight	x		x	x	x	x	x	
Privacy and security	x		x	x	x		x	x
Safety and wellbeing	x		x	x		x	x	
Responsibility			x	x	x	x	x	
Autonomy			x	x		x	x	x
Justice			x	x			x	
Transparency	x			x			x	

#### 3.2.1 Integration

Almost all documents mentioned that neuroethics must be integrated early in research and should be continually engaged throughout the process. Several of the documents also argued that, to ensure effectiveness, neuroethics efforts require support with sufficient resources (*BCI Report*, *NFS*, *GM1*, *GM2*, *NIH EENAS*, *BRAIN 2.0*). Some documents called for allocating no less than 5% of the total BRAIN Initiative budget to neuroethics efforts, which is in line with ELSI‐funding models from the Human Genome Project [28]. Moreover, two documents called for interagency partnership and the engagement of external experts to anticipate potential concerns and to develop ethically appropriate approaches and responses (*BCI Report*, *NFS*).

#### 3.2.2 Capacity Building and Strengthening (For Different Stakeholders)

Almost all documents recommended building neuroethics capacity and strengthening ongoing efforts. For example, some documents suggested that, to ensure integration and understanding of both neuroethics and neuroscience, experts in each respective field should be educated on the other, and that there should be tools and resources to facilitate such exchanges (*GM2*, *NIH EENAS*, *BRAIN 2.0*). These documents recommended the representation and active participation of neuroethicists on all neuroscience research teams. Moreover, several documents recommended that neuroethicists be represented in roles with various levels of project oversight authority, such as advisory body membership, etc. (*NFS*, *NIH EENAS*, *BRAIN 2.0*). Several documents recognized that this requires resource allocation for the establishment of training programs and management teams, as well as funding/grants in ethical neuroscience research (*NFS*, *GM1*, *NIH P*, *NIH EENAS*, *BRAIN 2.0*). Some documents also focused on prioritizing public education to enable fuller understanding of and engagement with neuroscience research (*NFS*, *GM1*, *NIH P*, *BRAIN 2.0*). Finally, the *Neurotechnology Futures Study* report recommended that research approaches and findings be communicated in a meaningful way to policy makers, legal experts, scholars, and the public to inspire engagement on potential ELSI, as well as mitigation strategies.

#### 3.2.3 Privacy and Security

Another relevant theme emerged around privacy and security. Several papers pointed out that brain‐derived data require confidentiality and privacy protections (*NIH P*, *NIH EENAS*, *BRAIN 2.0*). The *Neurotechnology Futures Study* report explicitly noted that the protection of such personal information could be achieved via amending the Health Insurance Portability and Accountability Act (HIPAA) Privacy Rule to also cover individually identifiable data obtained and used beyond the medical realm. Another paper pointed out that the BRAIN Initiative could establish guidelines governing the neuroscience data ecosystem, addressing data capture, storage, sharing, and translation (*NIH EENAS*). Similarly, the second volume of *Gray Matters* encouraged the adoption of educational tools aimed at legal scholars and practitioners to prevent misuse of neuroscience data in legal settings. Finally, the newly enacted Colorado Privacy Act amendment explicitly includes “neural data” within the protected types of sensitive personal data.

#### 3.2.4 Guidance and Oversight (Clinical/Biomedical and Beyond)

Several documents highlighted the need for policy guidance around neuroscience research and development, and use of neurotechnology (*NFS*, *GM2*, *NIH P*, *NIH EENAS*, *BRAIN 2.0*). Various documents specifically underscored the necessity of oversight mechanisms beyond those governing biomedical research and the clinical context (*NIH EENAS*, *BRAIN 2.0*). These calls extend to the legal setting (e.g., courtrooms) and neuromarketing (*GM1*, *BRAIN 2.0*). Several uses of neuroscience and neurotechnology that reach beyond biomedical and clinical applications are not covered by mechanisms governing NIH‐funded projects. In addition, one document called for advance planning for institutional implications in the context of BCI military applications (*BCI Report*).

#### 3.2.5 Safety and Wellbeing

Ensuring safe and appropriate use arose as a core theme in several documents (*NFS*, *GM2*, *NIH P*, *BRAIN 2.0*), and the *BCI Report* focused on military BCI applications, emphasizing safety risks. Most of the documents tagged the importance of safety and risk management as an imperative in the informed consent process. Two of the documents explicitly discussed the need to assess benefits and risks, with the help of neuroethicists, throughout the research process (*GM2*, *NIH P*), and the *Neurotechnology Futures Study* report noted the need to extend or revise existing regulations on safety and efficacy as new neurotechnologies emerge.

#### 3.2.6 Responsibility

The two BRAIN neuroethics subgroup reports highlighted a role for the BRAIN Initiative in jumpstarting conversations and collaborations around neuroscience applications beyond biomedical and clinical contexts (*NIH EENAS*, *BRAIN 2.0*). Other documents focused on: avoiding hype, overstatement, and unfounded conclusions (*GM2*, *BRAIN 2.0*); cautiously moving neuroscience tools and neurotechnologies into medical and nonmedical arenas (*NIH P*); attending to potentially malign uses of neuroscience tools and neurotechnologies (*NIH P*); considering and addressing posttrial responsibilities (*BRAIN 2.0*); and contending with the trust deficit among relevant groups (*BCI Report*).

#### 3.2.7 Autonomy

A clear need to define the consent capacity of neuroscience research participants emerged among the documents (*BCI Report*, *GM2*, *NIH P*, *BRAIN 2.0*). *Gray Matters Vol. 2* provides numerous recommendations focused on capacity to consent, including: responsibly involving participants with impaired capacity in neuroscience research, supporting research on capacity to consent, and ethical protections. RAND's *BCI Report* on military BCI applications also emphasizes the need for informed and voluntary consent.

#### 3.2.8 Justice

A few documents (*GM2*, *NIH P*) focused on justice by stressing the need to ensure equitable accessibility of neurotechnology while preventing exacerbation of existing social inequities. Relatedly, the *BRAIN 2.0* report specifically discussed posttrial responsibilities to participants in neurodevice clinical trials as a matter of social justice.

#### 3.2.9 Transparency

Transparency was acknowledged by three documents as critical to avoid unduly concerning the public. In particular, documents raising this theme called for mechanisms to ensure transparency (e.g., oversight mechanisms and encouraging public education and dialogue) so that the public can understand the risks and benefits of neuroscience research (*NFS*, *NIH P*, *BRAIN 2.0*), including, for example, more precise evidence on what types of neurotechnology are actually being developed for military national security purposes.

## 4 Discussion

Careful examination of key areas targeted by legislation and soft‐policy endeavors sheds light on potential regulatory gaps, issues garnering significant attention, and biases and preferences toward given governance mechanisms. Therefore, this analysis can inform policy makers about specific policy needs, while simultaneously encouraging the advancement of ethical and socially responsible neuroscience and neurotechnology innovation and deployment.

At present, the number of US policy documents related to neurotechnology remains limited. Excepting Colorado's Privacy Act amendment (enacted in April 2024) and California's amendment to the California Consumer Privacy Act (enacted in September 2024), at the time of analysis, only recommendations and suggestions exist—greatly limiting the authority of the identified governance documents. The recommendations are also either very broad or very narrowly applicable. As an example of limited applicability, the BRAIN Initiative‐linked reports applied their findings only to projects funded by the BRAIN Initiative; therefore, resulting recommendations are likely to have less impact on developments that extend beyond the scope of the NIH (i.e., the main sponsor of three identified documents). A similar illustrative example of narrow focus arises with regard to ethics integration: BRAIN Initiative grant mechanisms [29] focusing on neuroethics (e.g., requesting grant applicants to respond to a neuroethics implication section). While this is very welcome, it must be acknowledged that ethics integration must also include non‐BRAIN Initiative‐funded endeavors. Moreover, no designated percentage of total BRAIN Initiative funding has specifically been allocated to ELSI.

In comparing professional society calls for governance in genetics and genomics, where professional societies have been active in trying to influence oversight norms, we found no such involvement in neuroscience and neurotechnology governance at the national level in the US. International professional societies, however, are certainly active in this arena (e.g., IEEE SA P7700 Recommended Practice for the Responsible Design and Development of Neurotechnologies). But, this may soon change, especially with recent state‐level legal developments in states like Minnesota, which at the time of publication has followed Colorado and California's footsteps to consider specific neural data protections.

### 4.1 A Critical Movement in Emergent Neuroscience and Neurotech Law and Policy

As our review has demonstrated, the federal government in the United States has not acted to regulated the development of neurotechnology, which has left regulation to the states, several of which have adopted policies that cover neurotechnology. While federal laws such as HIPAA, which is critical to protect confidentiality of data from medical applications, and HITECH, which is critical to government protection of research and applications, provide examples of enforceable laws applicable to some neurotechnology, some state legislatures have enhanced the legal protections offered by such federal legislation and regulation. This has led to the establishment of state‐level rights related to “consumer health data” and application to areas typically outside the scope of HIPAA/HITECH. For example, the Washington My Health, My Data Act (MHMDA), while not drafted specifically for oversight of neurotechnology, applies to these technologies and the consumer health data they generate [30]. Enactment of Washington's MHMDA was followed promptly by legislative actions in other states, with Nevada (SB 370) and Connecticut (SB 3) passing similar laws [31, 32].

While technology‐agnostic efforts proceed, another approach gaining momentum is one proposing specific, narrowly‐focused legislation, including Colorado's amended Privacy Act, which, at the time, was touted as the “first‐in‐the‐nation neural data protections bill” [18]. California—a state recognized as leader in data privacy legislative reform with its comprehensive data protection laws (i.e., CCPA and CPRA)—amended its definition of “sensitive personal information” to explicitly include neural data [33]. But state‐level actions pose consistency repercussions, as well as potentially raise federal‐level implications in the future [34]. For example, such implications are currently being witnessed in the emerging patchwork of disparate abortion legislation [35].

Neurodata policies and regulation also has arisen at the international level [36, 37]. This is the case despite some scholars’ cautions that such policies are impractical [38, 39]. The European Union, with its General Data Protection Regulation (GDPR) (among other laws), is often seen as offering more robust data protections than the US. GDPR does not explicitly address neural data, although such data are reasonably expected to be protected within the “special categories of personal data” provision requiring heightened safeguard measures (such as specific consent and transparency) [40, 41]. Other key relevant provisions include the right to be forgotten and the right to approve transfer of personal data.

Indeed, legitimate concerns exist regarding the efficacy of such neuro‐specific legislative approaches. For instance, amending definitions to specifically enumerate neural data as a type of protected data might alter judicial interpretation of the statute in question. Similarly, insertion of neural data by amendment (such as the action taken in California) might cause judges to view the articulated data types as constituting an exhaustive list instead of an illustrative one. Such concerns have long plagued the federal Genetic Information Nondiscrimination Act (GINA), as questions swirl around whether GINA's definitions of “genetic information” or “genetic test” require amendment to clarify that they encompass epigenetic, microbiome, or other similar data, or, alternatively, whether coverage of such data is clarified via practice and caselaw. Similarly, the Americans with Disabilities Act (ADA) is relevant as neurotechnology can be used to determine evidence of neurological impairments, which could be the basis for discrimination; and just like GINA, the ADA contains ambiguities that could affect its application, such as what counts as a disability and how to best respond to technological innovations, such as those presented by neurotechnology.

Furthermore, the actual need for new, neurotech‐specific regulation in the United States remains an open question. For example, the role of the Federal Trade Commission (FTC) is often overlooked and underestimated in governing emerging technologies [42]. Whether direct‐to‐consumer genetic testing, mobile health apps [43], AI tools, or even neurotechnologies, the FTC holds nimble statutory authority to ensure basic consumer protections. While the FTC's enforcement mechanisms and oversight resources are notably constrained, the FTC has played a critical role in advancing responsible, technology‐agnostic practices intended to: (1) encourage innovation and entrepreneurship (via its focus on fair methods of competition); and (2) protect consumers from harms that might arise from unreasonable data privacy and security risks; deceptive data collection, use, and transfer practices; and unsubstantiated marketing claims [44, 45]. To promote competition, for example, the FTC can challenge mergers and acquisitions that consolidate and concentrate market power, block exclusive dealings among industry partners, and challenge aggressive “non‐compete” contracts that unfairly restrain neuroscientists from launching new companies, products, and services. To protect consumers, for example, the FTC can impel specific performance by neurotechnology developers to implement reasonable data privacy and security standards; to substantiate any claims made about health or cognitive benefits from their products; to provide clear, conspicuous, and “reasonably understandable” notices of data practices (including notices of data breaches); to obtain nonambiguous consent; and more. Though the FTC's activity might ultimately prove insufficient for long‐term oversight, the agency can provide invaluable short‐term protections while legislators consider new policies for governing specific technologies or use cases. Moreover, strong FTC governance might spark legislative activity—either to reinforce the direction FTC general policy would lead or to redirect specific policy in another direction or to another authority. State attorneys‐general can bolster federal efforts by the FTC by enforcing state‐level unfair and deceptive acts and practice statutes (akin to the Federal Trade Commission Act (15 U.S.C. §§ 41‐58, as amended)). Moreover, existing state and federal statutes and regulations (such as the recently updated FTC Health Breach Notification Rule (16 CFR Part 318) [46]) can be broadly interpreted to encompass neurotech data and scenarios.

### 4.2 Next Steps and Future Considerations

Governance of emergent technology is messy. Even if we agree that there is or can be some type or level of effective governance, it is often unclear at what point in the development and/or deployment of emergent technologies such oversight becomes necessary. For example, in translating genomic sequencing (i.e., WES and WGS), little governance existed—clinics simply started doing it [47]. With the current AI Bill of Rights and emerging state‐level legislative activity to regulate AI (see [48]), some lessons might emerge that are also applicable to the neuroscience and neurotechnology landscape. Notably, this is yet another area where Colorado has been moving quickly, as it just enacted the Colorado AI Act (SB‐205) [49].

Whether there is an actual need for neurotech‐specific law separate and apart from existing generally applicable laws (including but not limited to HIPAA/HITECH and the FTCA at the federal level) remains an open question at this juncture. Similarly, there is no broad consensus that adequate neurotech rights and neuroethics norms would be best achieved through an exceptionalist lens as opposed to a contextualist lens analogous to that advocated by Garrison and colleagues in regard to genetic/omic technologies [50]. Given the diversity of governance approaches (e.g., from constitutional amendments to soft‐governance mechanisms arising in the international arena), an informed, balanced public dialogue must occur in the near term. Moreover, governance endeavors must contemplate the themes identified by our analysis. For example, as neurotechnologies move into numerous domains of application, capacity building with relevant groups (e.g., judges, attorneys, military personnel, and many others) will serve as a critical foundation. In this context, the McArthur Foundation has established a relevant network and list of resources related to neurotech‐related ELSI in an effort to engage those in law‐related fields, but much more work remains to be done. Similarly, the work undertaken by IEEE BRAIN Neuroethics Framework offers an opportunity to examine neurotech‐related ELSI across nine different application domains, highlighting areas where little engagement and capacity building have occurred at present.

While several of the documents we identified and reviewed will likely be supersede as technology and law developed, they provide insight into how the governance and policy landscape of neurotechnology is evolving—and underscore that much work remains. As a result, we hope the findings set forth in this article will inform next steps.

## Conflicts of Interest

The authors declare no conflicts of interest.

## Endnotes

1 Existing reviews cover international soft policies, some of which include U.S. documents, but none with the specific focus we have for this review.

2 While several states have considered such bills (e.g., Connecticut, Massachusetts, Minnesota, Illinois, and Vermont), only Colorado had introduced and enacted a bill to protect neural data during the search period of our review (which ended in April 2024, the same month that the Colorado bill was signed into law). California signed a bill (S.B. 1223) into law in September 2024, amending the California Consumer Privacy Act's definition of “sensitive personal information” to explicitly include neural data. Montana, adopted SB 163 in May 2025, a law aimed at protecting mental privacy via regulating specific types of neurological data.
